# PHLDA2 reshapes the immune microenvironment and induces drug resistance in hepatocellular carcinoma

**DOI:** 10.32604/or.2024.047078

**Published:** 2024-05-23

**Authors:** KUN FENG, HAO PENG, QINGPENG LV, YEWEI ZHANG

**Affiliations:** 1 Hepatopancreatobiliary Center, The Second Affiliated Hospital of Nanjing Medical University, Nanjing, 210000, China; 2 Medical School, Southeast University, Nanjing, 210000, China

**Keywords:** HCC, Immune infiltration, Drug resistance, TME, PHLDA2

## Abstract

Hepatocellular carcinoma (HCC) is a malignancy known for its unfavorable prognosis. The dysregulation of the tumor microenvironment (TME) can affect the sensitivity to immunotherapy or chemotherapy, leading to treatment failure. The elucidation of PHLDA2’s involvement in HCC is imperative, and the clinical value of PHLDA2 is also underestimated. Here, bioinformatics analysis was performed in multiple cohorts to explore the phenotype and mechanism through which PHLDA2 may affect the progression of HCC. Then, the expression and function of PHLDA2 were examined via the qRT-PCR, Western Blot, and MTT assays. Our findings indicate a substantial upregulation of PHLDA2 in HCC, correlated with a poorer prognosis. The methylation levels of PHLDA2 were found to be lower in HCC tissues compared to normal liver tissues. Besides, noteworthy associations were observed between PHLDA2 expression and immune infiltration in HCC. In addition, PHLDA2 upregulation is closely associated with stemness features and immunotherapy or chemotherapy resistance in HCC. *In vitro* experiments showed that sorafenib or cisplatin significantly up-regulated PHLDA2 mRNA levels, and PHLDA2 knockdown markedly decreased the sensitivity of HCC cells to chemotherapy drugs. Meanwhile, we found that TGF-β induced the expression of PHLDA2 *in vitro*. The GSEA and *in vitro* experiment indicated that PHLDA2 may promote the HCC progression via activating the AKT signaling pathway. Our study revealed the novel role of PHLDA2 as an independent prognostic factor, which plays an essential role in TME remodeling and treatment resistance in HCC.

## Introduction

Hepatocellular carcinoma (HCC) comprises approximately 90% of primary hepatic malignancies and is frequently derived from hepatic progenitor cells or hepatocytes [1,2]. It is reported that HCC is predicted to be the sixth most frequently diagnosed cancer and the fourth leading cause of cancer deaths worldwide [3]. Although the early screening and diagnostic methods of HCC have made significant progress, the prognosis is still unsatisfactory. A cumulative HCC recurrence after surgery is as high as 70% at five years [4]. Moreover, molecular profiling still cannot predict the outcomes of patients and the risk of recurrence after successful surgery or ablation [5]. Thus, additional biomarkers are urgently needed to improve prognosis or predict treatment response.

The significance of the tumor microenvironment (TME) in tumor development is gradually being discovered. The constituent characteristics of TME include immune cells, stromal cells, blood vessels, and extracellular matrix, in which various adaptive and innate immune cells can perform tumor-promoting or tumor-suppressive functions [6]. In addition, cancer stem cells (CSCs) exhibit stemness characteristics that contribute to tumor progression, metastasis, recurrence, and drug resistance. Moreover, the complex interaction network between CSCs and immune cells further aggravates the difficulty of treatment [7]. Accumulating studies have also shown that the dysregulation of the immune microenvironment promotes the occurrence of drug resistance in tumors [8,9].

Pleckstrin-homology-like domain family A member 2 (PHLDA2), previously known as TSSC3 and IPL, is a cluster of imprinted genes located on human chromosome 11p15.5 and is expressed by maternal alleles during normal developmental processes [10,11]. Moreover, PHLDA2 was the first identified apoptosis-related imprinted gene that regulates placental growth [12]. In addition, PHLDA2 has been observed to display abnormal expression patterns in several malignant tumors, such as glioma, hydatidiform mole, osteosarcoma, and colorectal cancer [13–16]. However, the role of PHLDA2 in HCC has yet to be clearly defined.

This study mainly explored the expression, prognostic value, and association between PHLDA2 and genomic variations in HCC. Meanwhile, the association between PHLDA2 and various immune cells within the immune microenvironment was determined. Through the enrichment analysis of PHLDA2-related genes, the biological functions and pathways PHLDA2 involved were explored. We also found that PHLDA2 was significantly associated with tumor stemness and involved in regulating drug resistance.

## Materials and Methods

### Data collection

The transcriptional expression profile, methylation, mutation, copy number variation, and clinical information of liver hepatocellular carcinoma (LIHC) were downloaded from The Cancer Genome Atlas (TCGA) database (https://portal.gdc.cancer.gov/) for further analysis. The expression of PHDLA2 across multiple cancer types was acquired from TIMER2.0 (http://timer.cistrome.org/). The other expression data and relevant clinical information were derived from the HCCDB (http://lifeome.net/database/hccdb/home.html). Additional data, such as GSE121153, were downloaded from the Gene Expression Omnibus (GEO) database (https://www.ncbi.nlm.nih.gov/geo/). Single-cell data analysis was obtained from the TISCH database (http://tisch.comp-genomics.org/).

### Prognostic analysis and nomogram

To evaluate the prognostic capability of PHLDA2 in HCC, Kaplan-Meier (KM) curves were generated for overall survival (OS), disease-specific survival (DSS), and progress-free interval (PFI) using clinical data derived from the TCGA LIHC cohort and the HCCDB6 cohort by the package “survminer” and “survival” in R. Besides, the package “survival” was used for proportional hazards hypothesis testing and Cox regression analysis. The independent prognostic value of PHLDA2 was evaluated through univariate and multivariate analyses. A nomogram was constructed based on the multivariate analysis by the package “survival” and “rms.” A calibration plot was employed to assess the performance of the nomogram, with a closer alignment between the predicted model line and the diagonal line indicating a better fit. The diagnostic receiver operating characteristic (ROC) analysis and test were conducted using the R package “pROC.”

### Genomic alterations and heterogeneity

The R package “maftools” presented the mutation landscape across various subgroups. Furthermore, the inferHeterogeneity function of the R package “maftools” was employed to calculate each sample’s mutant-allele tumor heterogeneity (MATH). The ploidy, loss of heterozygosity (LOH), and homologous recombination deficiency (HRD) were obtained from previous studies [17].

### Analysis of immune infiltration

The XCELL, MCPCOUNTER, CIBERSORT, TIMER, EPIC, and QUANTISEQ algorithms, which utilize transcriptome data, were employed to examine the composition and proportion of various immune cells in HCC tissues within the TIMER2.0 database [18]. The R package “ESTIMATE” was utilized to compute stromal cells and immune cells in tumor tissue. The immune and molecular subtypes were derived from the TISIDB database (http://cis.hku.hk/TISIDB/).

### Biological enrichment

The differentially expressed genes (DEGs) were identified through the R package “limma” between different subgroups, with a threshold of |log_2_FC| > 1 and p.adj < 0.05. Subsequently, the R packages “ClusterProfiler” and “org.Hs.eg.db” were used to analyze pathway enrichment for Gene Ontology (GO) and Kyoto Encyclopedia of Genes and Gene Ontology (KEGG). The significance of enrichment of DEGs in pre-defined gene sets, such as the Hallmark gene set, was evaluated using Gene Set Enrichment Analysis (GSEA) to assess their contribution to the phenotype. The expression matrix was converted into a gene set enrichment score matrix using Single-Sample Gene Set Enrichment Analysis (ssGSEA) with the R package “GSVA” to obtain the enrichment score of each gene set in each sample. The EMT-related signatures were obtained from the EMTome (http://www.emtome.org/).

### Stemness analysis

Consensus clustering was applied to identify distinct stemness-related clusters based on stemness-related gene sets by the R package “ConsensusClusterPlus”—the procedure calculated Euclidean distance with a km approach. The maximum number of clusters was 6, and 80% of the sample was drawn 1000 times. The three proven stemness-related gene sets were downloaded from the MSigDB (https://www.gsea-msigdb.org/gsea/msigdb/), including “YAMASHITA LIVER CANCER STEM CELL UP” (YAMASHITA UP), “YAMASHITA LIVER CANCER STEM CELL DN” (YAMASHITA DN), and “WONG EMBRYONIC STEM CELL CORE” (WONG CORE). A unique gene set was derived from a previous study [19].

### Estimation of immunotherapy and chemotherapy

The ImmuneCellAI algorithm (http://bioinfo.life.hust.edu.cn/ImmuCellAI#!/) was used to predict the immunotherapy response in different patients. The Tumor Immune Dysfunction and Exclusion (TIDE) algorithm (http://tide.dfci.harvard.edu/) was also used to verify responders and non-responders. Besides, the TIDE algorithm can provide a TIDE score and MSI Expr Sig [20]. The R package “oncoPredict” was used to predict the drug sensibility.

### Cell culture and transfection

The human liver cancer cell lines HepG2, Hep3B, and SMMC-7721, as well as the normal liver cell line LO2, were cultured in Dulbecco’s modified Eagle’s medium (DMEM) supplemented with 10% fetal bovine serum and 1% penicillin-streptomycin (Beyotime, China). The cultures were maintained at 37°C and 5% carbon dioxide. The DMEM and fetal bovine serum were obtained from Gibco, Thermo Fisher Scientific, in the United States. The PHLDA2-specific small interfering RNAs (siRNAs) and negative control (NC) were designed from the Generay (Shanghai, China). The sequences of siRNA-1 (si-1) were 5′-GGCAAGUACGUGUACUUCATT-3′ (F), 5′-UGAAGUACACGUACUUGCCTT-3′ (R), and the sequences of siRNA-2 (si-2) were 5′-GCUUCCACUCCAUCCUCAATT-3′ (F), 5′-UUGAGGAUGGAGUGGAAGCTT-3′ (R). Cell transfection was performed using Lipo8000™ Transaction Reagent (Beyotime, China) in 6-well cell culture plates (LABSELECT, China).

### RNA isolation and quantitative RT-PCR

Total RNA was extracted using a total RNA isolation reagent (Biosharp, China), and the reverse transcription was carried out using SweScript RT I first strand cDNA synthesis Kit (Servicebio, China) to obtain cDNA. Then, quantitative RT-PCR (qRT-PCR) was performed with 2 x SYBR Green qPCR Master Mix (High ROX) (Servicebio, China). The CT value was detected by a real-time PCR system (Kubo, China). All were operated according to the kit instructions. GAPDH was employed as an internal reference control, and the primer sequences were 5′-GGAGCGAGATCCCTCCAAAAT-3′ (F), 5′-GGCTGTTGTCATACTTCTCATGG-3′ (R). The primer sequences of the target gene PHLDA2 were 5′-CGACAGCCTCTTCCAGCTAT-3′ (F), 5′-CAGCGGAAGTCGATCTCCTT-3′ (R). The primers were synthesized at Springen (Nanjing, China).

### Protein extraction and western blotting

RIPA lysis buffer (Beyotime, China) was used for cellular protein extraction. Equal amounts of protein were separated by SDS-PAGE (Beyotime, China) and transferred onto a PVDF membrane (Merck, Darmstadt, Germany), then blocked with 5% bovine serum albumin (BSA) (Servicebio, China) in tris-buffered saline (Servicebio, China) for one hour at room temperature. The membrane was subjected to incubation with specific primary antibodies at a temperature of 4°C for the duration of one night, after which it underwent incubation with suitable secondary antibodies conjugated with horseradish peroxidase (Servicebio, China) for one hour at ambient temperature. The signals were subsequently detected by employing an enhanced chemiluminescence reagent (Beyotime, China) and subjected to chemiluminescent detection (Tanon, Shanghai, China). The primary antibodies for GAPDH, AKT, and phosphorylated-AKT (p-AKT) were purchased from the Cell Signaling (Massachusetts, USA).

### MTT assay

Transfected cells were distributed into 96-well plates at a density of 4000 cells per well. Following overnight adherence, the culture medium was substituted with 100 μl of fresh medium containing varying concentrations of sorafenib (Macklin, Shanghai, China). The cells were then cultured for either 24 or 48 h. Subsequently, 5 mg/ml of MTT (Beyotime, China) was introduced to each well and incubated for 4 h. The supernatant was discarded, 150 μL DMSO (Beyotime, China) was added, and a microplate reader (ALLSHENG, Hangzhou, China) measured the OD value at 490 nm.

### Statistics

The expression levels and scores between the two groups were examined using the Wilcoxon test. The Spearman correlation test was employed to conduct correlation analyses. The one-way ANOVA and Kruskal-Wallis tests were utilized to compare the difference between more than two groups. A *t*-test was employed to assess the disparities between the two groups for the experimental data. Each experiment was performed three times. Statistical significance was as follows: ns, not significant; *p < 0.05; **p < 0.01; ***p < 0.001; ****p < 0.0001.

## Results

### The expression and prognosis value of PHLDA2 in HCC

**Figure 1:**
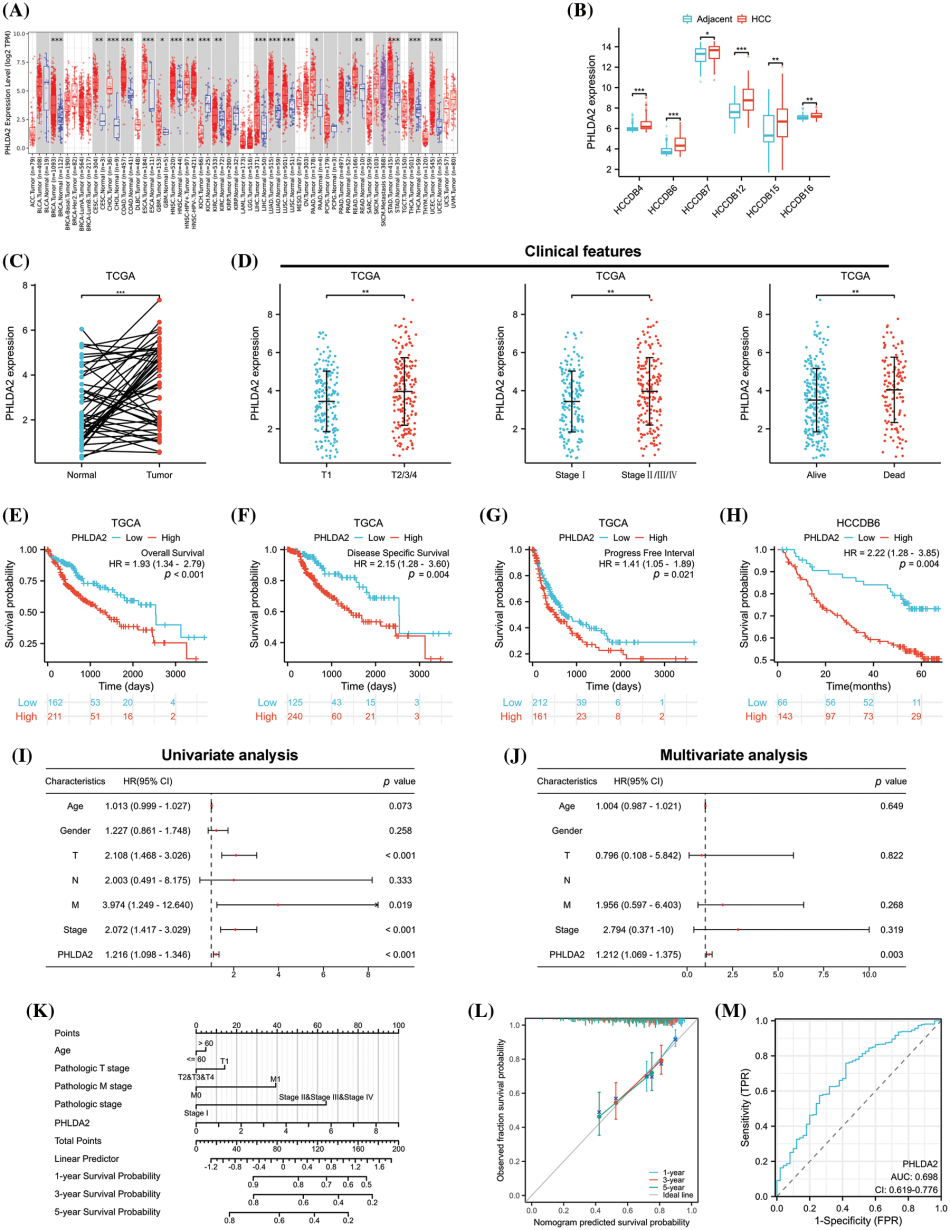
The expression and prognostic value of PHLDA2 in HCC. (A) The expression of PHLDA2 in various malignant tumors and normal tissues in the TIMER2.0. (B) The expression of PHLDA2 in HCC and liver tissues from the HCCDB. (C) PHLDA2 expression in HCC compared to adjacent liver tissues in the TCGA. (D) The relationships between PHLDA2 and clinical characteristics in HCC, including T stage, stage, and status. (E–H) Kaplan–Meier analysis for PHLDA2 in the TCGA and HCCDB-6 cohorts. (I, J) The forest plot for univariate and multivariate analysis of PHLDA2 and clinical features for OS from the TCGA. (K) The nomogram plot based on PHLDA2 and clinicopathological factors for OS. (L) The calibration plot for nomogram validation. (M) The diagnostic ROC curve for PHLDA2. *p < 0.05; **p < 0.01; ***p < 0.001.

To determine the expression of PHLDA2 in both tumor and normal tissues, we conducted a query of the TIMER database. The results indicated that PHLDA2 exhibited a high level of expression in 16 different cancer types (Fig. 1A), including BRCA, CESC, CHOL, COAD, ESCA, GBM, HNSC, KIRC, LIHC, LUAD, LUSC, PAAD, READ, STAD, THCA and UCEC. Then, we searched PHLDA2 expression specifically in hepatocellular carcinoma (HCC) using the HCCDB database. Our findings revealed that the expression level of PHLDA2 was significantly elevated in HCC tissues compared to adjacent liver tissues across multiple cohorts (Fig. 1B). According to the TCGA database, the expression of PHLDA2 was similarly higher in HCC tissues compared to paired liver tissues (Fig. 1C). Furthermore, the correlation between PHLDA2 and clinical features was also explored. The clinical features of HCC patients are presented in Table 1. The PHLDA2 expression was higher in patients with T2/3/4, Stages II/III/IV, and status dead (Fig. 1D). We also investigated the significance of PHLDA2 in the prognosis and diagnosis of HCC patients. The findings indicated a potential association between elevated PHLDA2 expression and a poorer prognosis in HCC patients. In the TCGA cohort, the PHLDA2-high HCC patients had a relatively shorter OS, DSS, and PFI (Figs. 1E–1G). Consistent with these results, we verified the effect of PHLDA2 on HCC prognosis in the HCCDB6 cohort (Fig. 1H). Based on univariate and multivariate Cox regression analysis, PHLDA2 was found to be the only independent risk factor for HCC patients (Figs. 1I–1J). Moreover, a nomogram including PHLDA2 and multiple clinicopathological factors was generated, which provided a semi-quantitative technique for the prognosis of patients (Fig. 1K). The calibration curve demonstrated a satisfactory alignment between actual and nomogram-predicted survival outcomes (Fig. 1L). The diagnostic ROC curve of PHLDA2 also revealed the value of PHLDA2 in the diagnosis of HCC, with the area under curve (AUC) = 0.698 (Fig. 1M). The findings above indicate a significant upregulation of PHLDA2 in hepatocellular carcinoma (HCC), which is associated with a poorer prognosis.

**Table 1:** The associations between PHLDA2 and clinical features of HCC patients from the TCGA

Characteristics	Low expression	High expression	*p* value
Age			0.719
<=60	87 (23.3%)	90 (24.1%)	
>60	100 (26.8%)	96 (25.7%)	
Gender			0.581
Female	63 (16.8%)	58 (15.5%)	
Male	124 (33.2%)	129 (34.5%)	
Pathologic T stage			0.647
T1	96 (25.9%)	87 (23.5%)	
T2	46 (12.4%)	49 (13.2%)	
T3	37 (10%)	43 (11.6%)	
T4	5 (1.3%)	8 (2.2%)	
Pathologic N stage			0.169
N0	120 (46.5%)	134 (51.9%)	
N1	0 (0%)	4 (1.6%)	
Pathologic M stage			0.159
M0	129 (47.4%)	139 (51.1%)	
M1	0 (0%)	4 (1.5%)	
Pathologic stage			0.089
Stage I	91 (26%)	82 (23.4%)	
Stage II	45 (12.9%)	42 (12%)	
Stage III	38 (10.9%)	47 (13.4%)	
Stage IV	0 (0%)	5 (1.4%)	
Histologic grade			0.042
G1	36 (9.8%)	19 (5.1%)	
G2	89 (24.1%)	89 (24.1%)	
G3	53 (14.4%)	71 (19.2%)	
G4	5 (1.4%)	7 (1.9%)	
Adjacent hepatic tissue inflammation			0.046
None	73 (30.8%)	45 (19%)	
Mild	46 (19.4%)	55 (23.2%)	
Severe	11 (4.6%)	7 (3%)	
BMI			0.026
<=25	78 (23.1%)	99 (29.4%)	
>25	90 (26.7%)	70 (20.8%)	

### The methylation levels of PHLDA2 and genomic alterations in HCC

**Figure 2:**
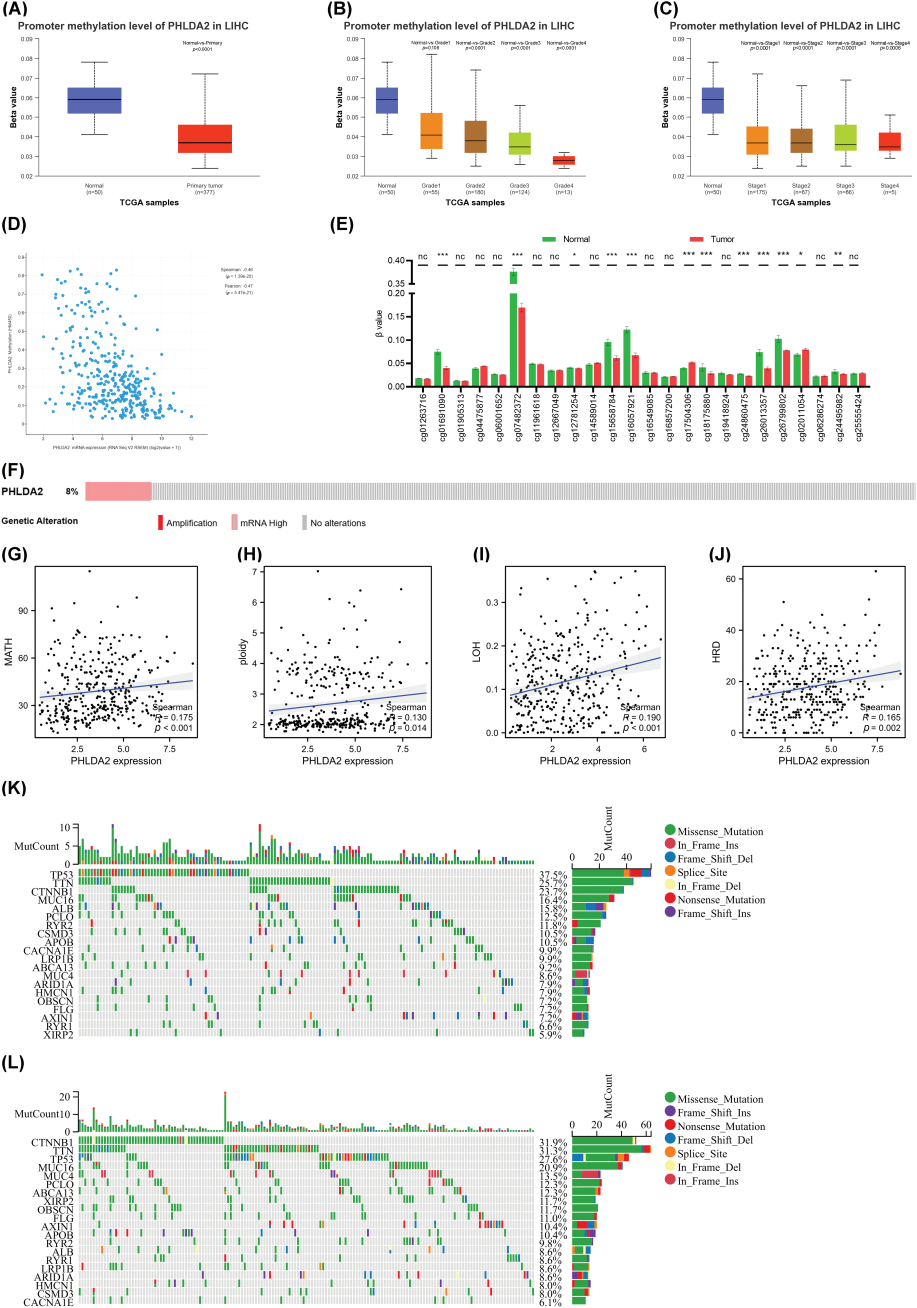
Analysis of methylation and genetic heterogenicity in HCC. (A) The promoter methylation level of PHLDA2 in HCC from the UALCAN. (B, C) The promoter methylation of PHLDA2 in HCC of various tumor stages and tumor grade by the UALCAN. (D) The relationship between the PHLDA2 and methylation in the cBioPortal. (E) The methylation of CpG islands in the PHLDA2 promoter region from the TCGA. (F) The genomic alterations of PHLDA2 in TCGA-LIHC from the cBioPortal. (G–J) The correlations between the PHLDA2 and MATH, ploidy, LOH, and HRD. (K, L) The somatic landscape of HCC in PHLDA2-low and PHLDA2-high subgroups. *p < 0.05; **p < 0.01; ***p < 0.001.

Then, we explore the regulatory factors of PHLDA2 overexpression in HCC. The methylation levels in the promoter region of PHLDA2 were first analyzed. Our analysis reveals considerably lower levels of PHLDA2 promoter methylation in HCC tissues compared to normal liver tissues through the UALCAN database (Fig. 2A). Moreover, the PHLDA2 promoter methylation levels were reduced in advanced stages and grades (Figs. 2B and 2C). Likewise, the expression of PHLDA2 also showed a negative correlation with methylation level based on the cBioPortal database (Fig. 2D). We further analyzed the methylation level of CpG islands located in the promoter region of PHLDA2 (region from −1500 nucleotide upstream to the transcription start site) in the TCGA database, and the results showed that the methylation level of PHLDA2 was lower in HCC tissues than in normal liver tissues (Fig. 2E). These results suggest that the elevated expression of PHLDA2 in HCC may be influenced by methylation modifications. Next, the alterations of PHLDA2 was further explored. PHLDA2 had 8% genetic alternations, including amplification and mRNA high, according to the cBioPortal database (Fig. 2F). Besides, we also analyzed the relationship between PHLDA2 and MATH, ploidy, LOH, and HRD, which are common indicators of genomic heterogeneity (Figs. 2G–2J). The results revealed that PHLDA2 was positively correlated with them. Then, the different mutational landscapes between the PHLDA2-low and PHLDA2-high subgroups were also compared. In the high PHLDA2 expression subgroup, TP53, an important tumor suppressor gene, is more prone to mutations, with a 37.5% mutation rate. However, CTNNB1, a key downstream component of typical WNT signaling pathways, has a higher mutation frequency, with a 31.9% mutation rate in the low PHLDA2 expression subgroup (Figs. 2K and 2L). These results suggested that the increased expression of PHLDA2 in HCC is prone to genomic mutations, leading to genomic instability, which may further lead to the occurrence and development of HCC.

### PHLDA2 is correlated with immune cell infiltration in HCC

**Figure 3:**
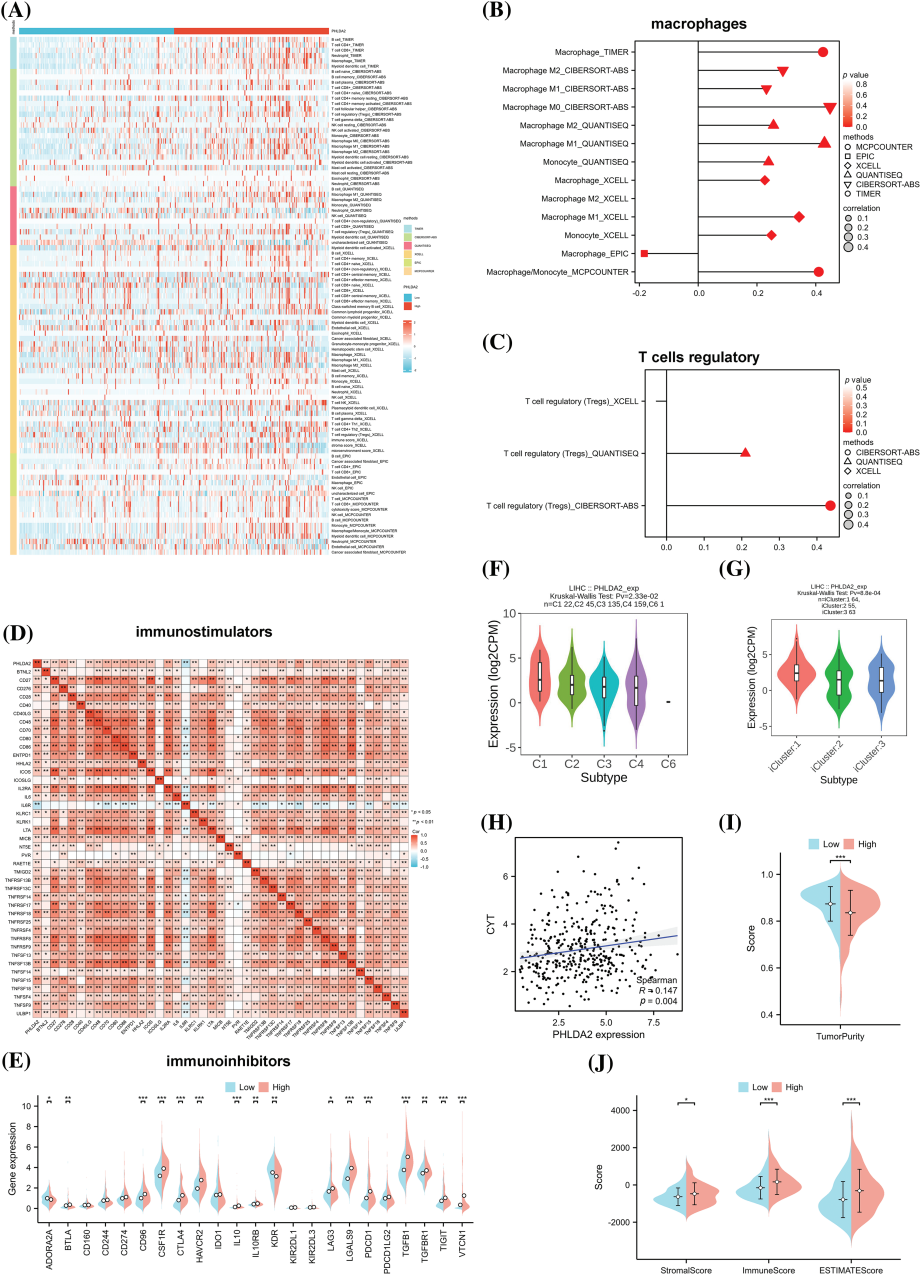
Correlation of PHLDA2 with immune infiltration in HCC. (A) The heat map of immune cell infiltration in PHLDA2-low and PHLDA2-high subgroups by the TIMER. (B, C) The lollipop graph showed the correlation between the PHLDA2, macrophages, and Tregs. (D) The Spearman correlation among PHLDA2 and several immunostimulators. (E) The expression of immunoinhibitors between PHLDA2-low and PHLDA2-high subgroup. (F, G) PHLDA2 expression in different immune subtypes and molecular subtypes from the TISIDB. (H) The correlation between CYT and the PHLDA2. (I) The violin plot showed the Tumor Purity in the PHLDA2-low and PHLDA2-high subgroups. (J) The StromalScore, ImmuneScore, and EstimateScore in PHLDA2-low and PHLDA2-high subgroup by ESTIMATE. *p < 0.05; **p < 0.01; ***p < 0.001.

Next, we explored the relationship between the PHLDA2 expression and immune cells infiltration. Evidence has revealed that the tumor immune microenvironment plays a crucial role in tumor progression [21]. Based on XCELL, MCPCOUNTER, CIBERSORT, TIMER, EPIC, and QUANTISEQ algorithms, we obtained the composition and proportion of various immune cells in the tumor immune microenvironment of HCC in the TCGA cohort. The construction of immune cell infiltration patterns differed in the PHLDA2 low and high subgroups, and the PHLDA2 high expression subgroup showed more abundant immune cell infiltration (Fig. 3A). The correlation analysis showed that PHLDA2 could increase the infiltration levels of myeloid dendritic cells, macrophage cells, T cells regulatory (Tregs), T cells CD4+, T cells CD8+, B cells, and NK cells in HCC, and PHLDA2 would reduce the proportion of endothelial cells (Figs. 3B and 3C; Suppl. Fig. S1). The Single-cell data also suggested that PHLDA2 was associated with various immune cells (Suppl. Fig. S2). In addition, we analyzed the relationship between PHLDA2 expression and immune-related molecules. The heatmap showed that PHLDA2 was positively correlated with most immunostimulatory molecules in HCC (Fig. 3D). Besides, the violin chart showed that the expression of most immunosuppressive molecules was higher in the PHLDA2-high subgroup (Fig. 3E). Furthermore, we found that there existed differential expression of PHLDA2 in different immune and molecular subtypes according to the TISIDB database, and the expression of PHLDA2 was higher in wound healing (C1) and icluster1 (Figs. 3F and 3G). C1 had elevated expression of angiogenic genes and a high proliferation rate based on previous research. Besides, the cytolytic activity score (CYT) is a well-established biomarker that predicts the presence of tumor-infiltrating lymphocytes based on gene expression [22]. We observed that the CYT score positively correlated with PHLDA2 expression in HCC tissues (Fig. 3H). Moreover, the Stromal, Immune, and ESTIMATE Scores obtained by the ESTIMATE algorithm also differed between the PHLDA2-low and PHLDA2-high subgroups. The scores were higher, obviously, in the PHLDA2-high subgroup. In contrast, the tumor purity was lower in the PHLDA2-high subgroup (Figs. 3I and 3J). These results indicated that PHLDA2 could recruit more immune cell infiltration to remodel the immune microenvironment of HCC.

### Elucidation of the potential functions and molecular mechanisms of PHLDA2 in HCC

**Figure 4:**
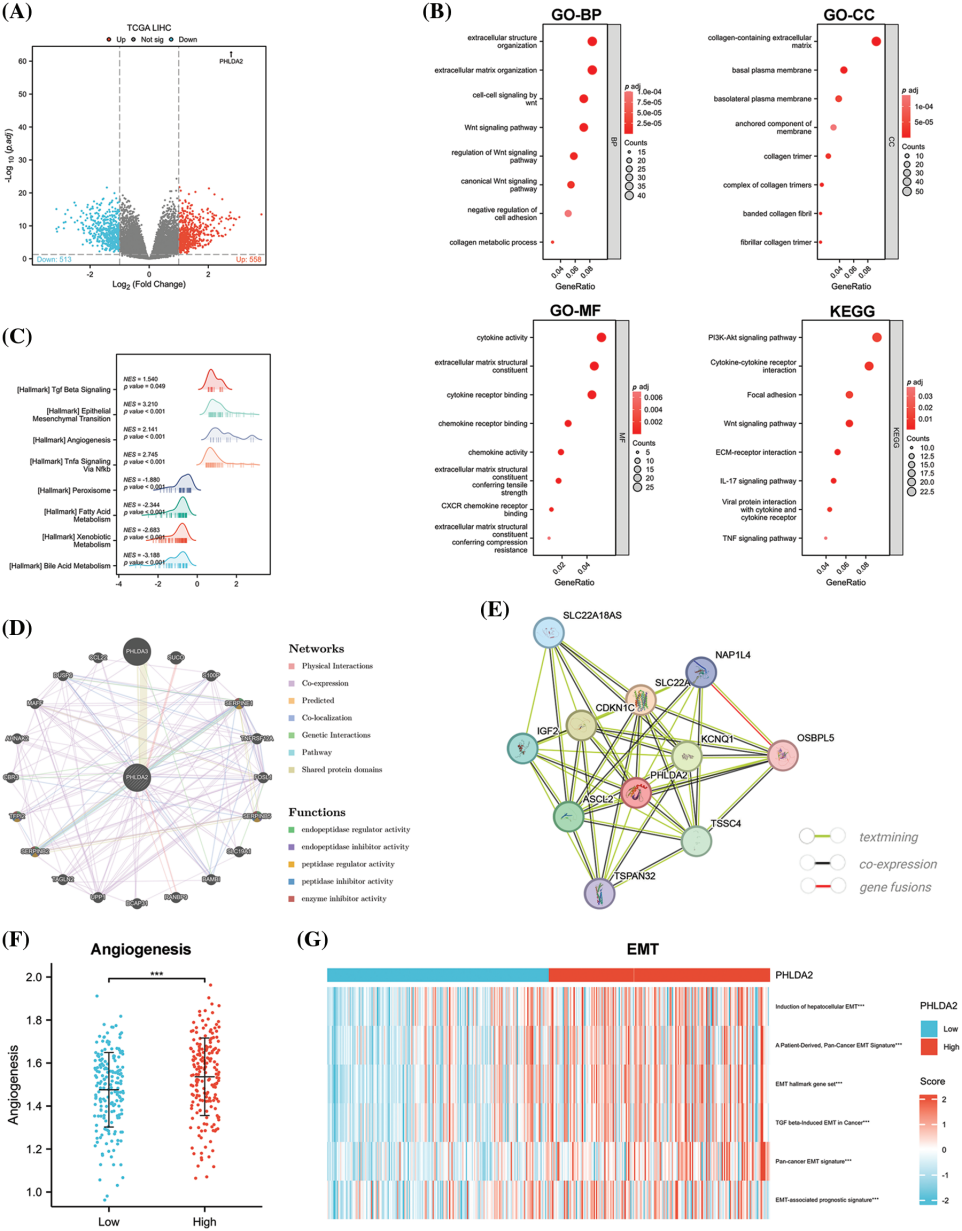
PHLDA2-related genes and functional enrichment analysis. (A) The enhanced volcano plot of differential genes between PHLDA2-high and PHLDA2-low subgroups. (B) Functional enrichment analysis by GO and KEGG. BP, Biological Process; CC, Cellular Component; MF, Molecular Function. (C) GSEA analysis of Hallmark gene set. (D, E) The protein-protein interaction networks of PHLDA2 from the Gene MANIA and STRING database. (F) Angiogenesis score between PHLDA2-high and PHLDA2-low subgroup. (G) The heat map of the EMT signature is in the PHLDA2-high and PHLDA2-low subgroups. ***p < 0.001.

And next, to explore the underlying signaling pathways and functional mechanisms that PHLDA2 may participate in, we firstly identified 513 down-regulated and 558 up-regulated DEGs between patients with high and low expression of PHLDA2 in the TCGA database (Fig. 4A). The GO and KEGG pathway enrichment analysis was then performed based on the up-regulated genes. The results showed that these genes were primarily involved in the WNT signaling pathway, PI3K-AKT signaling pathway, TNF signaling pathway, extracellular matrix organization (ECM), cytokine activity, and chemokine activity, etc. (Fig. 4B). The GSEA analysis of the Hallmark gene set was mainly enriched in terms of the epithelial-mesenchymal transition (EMT), angiogenesis, TNF signaling via NF-κB, and TGF-β signaling, etc. (Fig. 4C). In addition, we constructed the interaction network of co-expressed genes and the protein-protein interaction networks of PHLDA2 through the GeneMANIA and STRING databases (Figs. 4D and 4E). Genes co-expressed with PHLDA2, such as SERPINE1, SERPINB5, SERPINB2, and TFPI2, were primarily involved in endopeptidase regulator activity and peptidase regulator activity from the GeneMANIA database. We also searched for the signature of angiogenesis on the MSigDB database and used the ssGSEA method to calculate the enrichment score. We found that the PHLDA2-high expression group had higher scores, consistent with the above results (Fig. 4F). Furthermore, we also found several EMT-related signatures from the EMTome database and used the ssGSEA method to calculate the score. The results showed that the PHLDA2-high expression subgroup also had higher scores (Fig. 4G). In summary, enrichment analysis results suggested that PHLDA2 may play a role in promoting tumor development by angiogenesis and EMT, and PHLDA2 may be involved in the regulation of the WNT signaling pathway, PI3K-AKT signaling pathway, TNF signaling pathway, and TGF-β signaling pathway.

### PHLDA2 is associated with stemness phenotype

**Figure 5:**
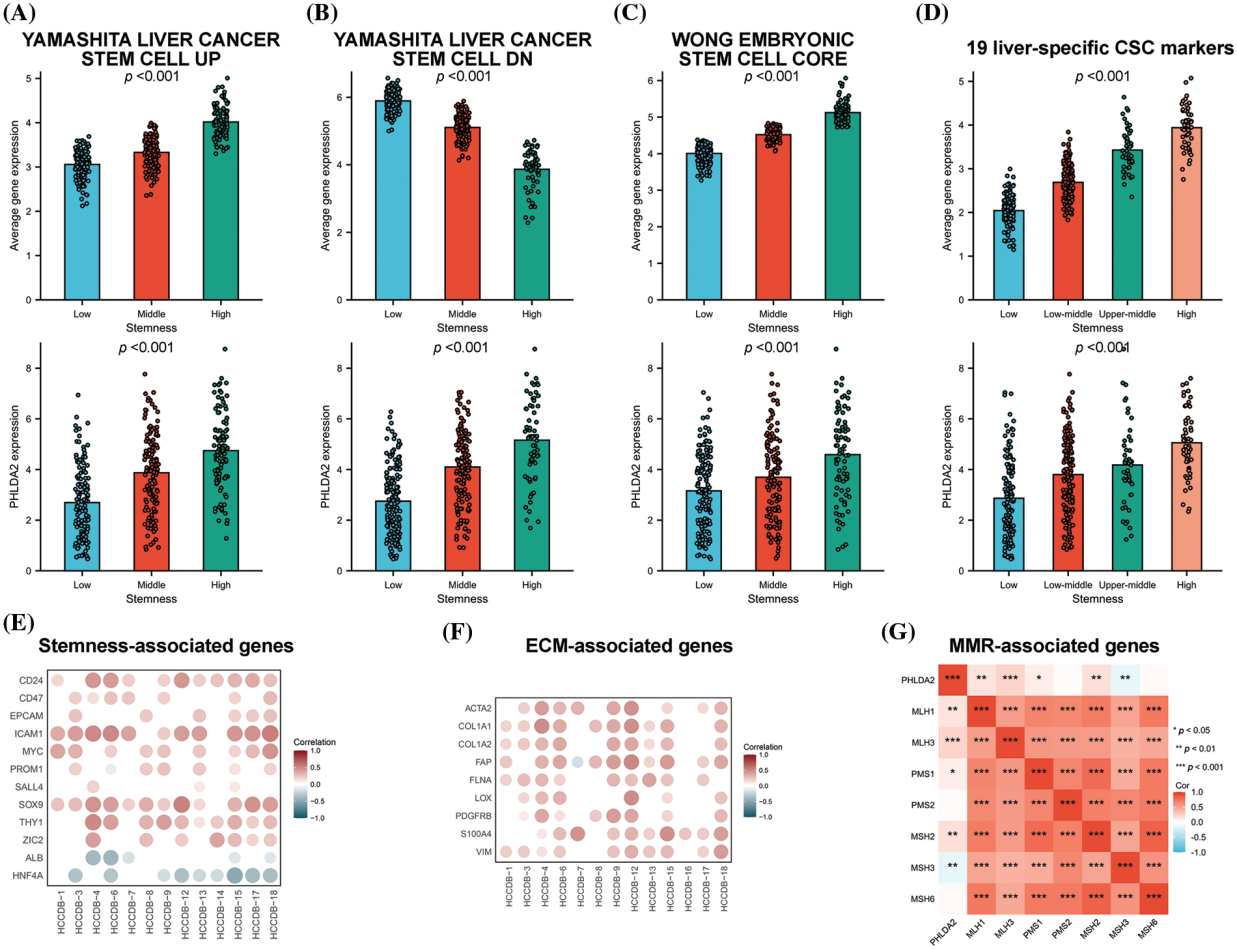
PHLDA2 is related to stemness characteristics. (A–D) The average expression level of the gene set and PHLDA2 in subgroups identified by the YAMASHITA UP gene set (A), the YAMASHITA DN gene set (B), the WONG CORE gene set (C), and 19 liver CSC markers (D). (E, F) The correlations between the PHLDA2 expression and stem-related genes (E) and ECM-relative genes (F) expression in the HCCDB database. (G) The correlation heat map of PHLDA2 and MMR-related gene expression. *p < 0.05; **p < 0.01; ***p < 0.001.

And then we examine the association between PHLDA2 expression and the stemness phenotype in HCC. Three established gene sets on stemness were obtained from the MSigDB database. A specific stemness gene set linked to HCC was also identified based on a prior investigation [19]. Subsequently, consensus clustering was executed on the TCGA cohorts using the unsupervised K-means consensus clustering method, wherein the YAMASHITA UP gene set revealed the presence of three stable subgroups exhibiting enhanced stemness. The average expression of genes in the YAMASHITA UP gene set was elevated with increased stemness. The expression of PHLDA2 was also promoted with increased stemness (Fig. 5A). Comparable outcomes were observed when employing the YAMASHITA DN gene set, WONG core gene set, and a specific gene set comprising 19 stemness-related genes (Figs. 5B–5D). We also analyzed the correlation between PHLDA2 and stemness-related genes in the HCCDB database. As expected, PHLDA2 showed a positive correlation with CD24, CD47, EPCAM, ICAM1, MYC, PROM1, SALL4, SOX9, THY1, and ZIC2, all of which were positively associated with stemness. Conversely, PHLDA2 exhibited a negative correlation with ALB and HNF4A, both of which were negatively associated with stemness (Fig. 5E). At the same time, we conducted an analysis on the relationship between PHLDA2 and ECM-related genes, including ACTA2, COL1A1, COL1A2, FAP, FLNA, LOX, PDGFRB, S100A4, and VIM, revealing a positive correlation between them (Fig. 5F). According to previous studies, DNA mismatch repair (MMR) also contributed to cancer stemness maintenance [23,24]. Hence, the correlation analysis between PHLDA2 and MMR-related genes was performed, and the result showed a positive correlation between PHLDA2 and MMR-related genes, including MLH1, MLH3, PMS1, and MSH2 (Fig. 5G). In all, PHLDA2 may play an essential role in tumor stemness maintenance.

### PHLDA2 could predict responses to immunotherapy and chemotherapy

**Figure 6:**
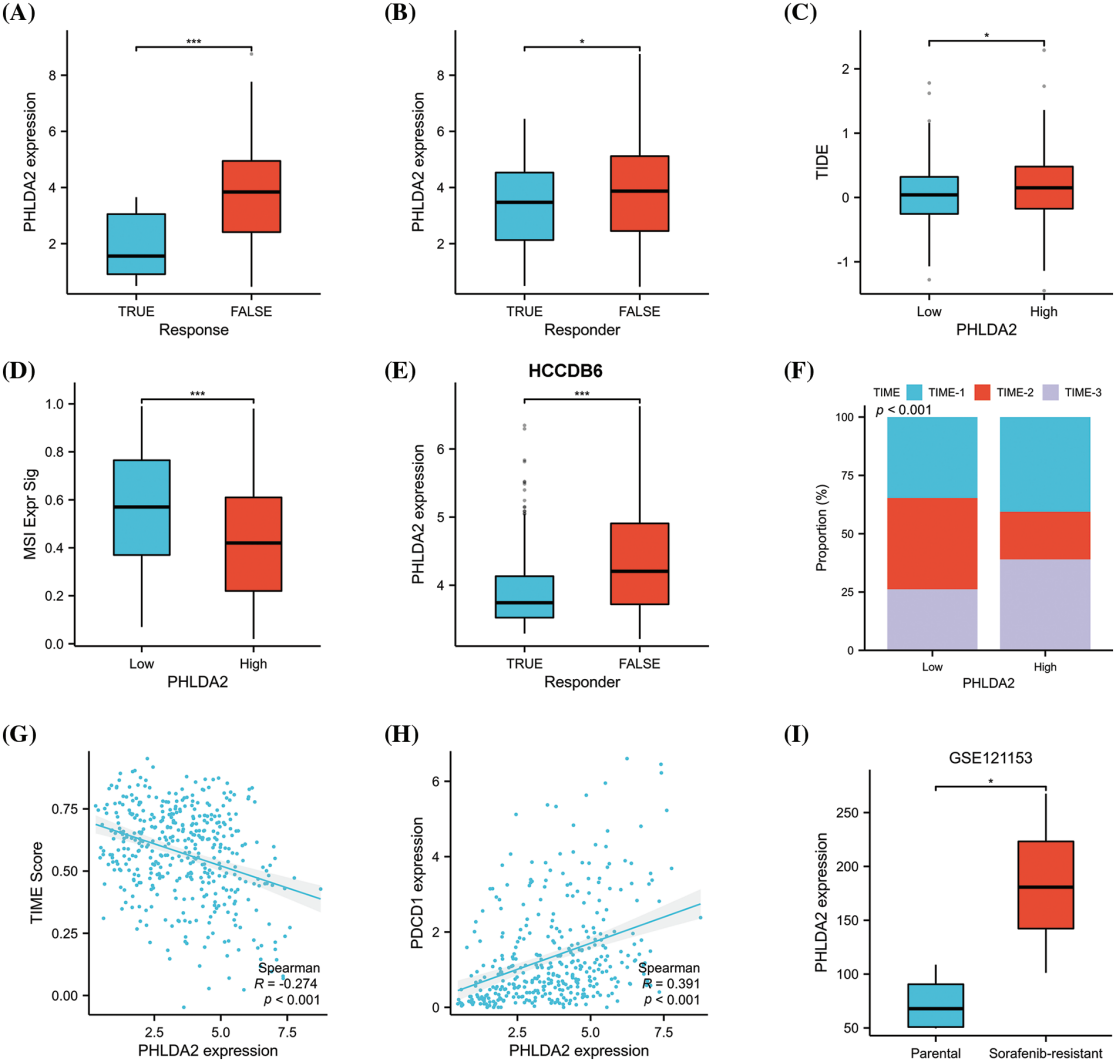
PHLDA2 could predict the clinical benefits of immunotherapy and chemotherapy. (A, B) PHLDA2 expression between responder or non-responder patients according to the ImmuCellAI (A) or TIDE (B) algorithm in the TCGA cohort. (C) The TIDE score is between the PHLDA2-low and PHLDA2-high subgroups. (D) The MSI signature score between the PHLDA2-low and PHLDA2-high subgroup. (E) PHLDA2 expression between responder or non-responder patients according to the TIDE algorithm in the HCCDB6 cohort. (F) The distribution of TIME subtypes in the PHLDA2-low and PHLDA2-high subgroups. (G) The scatter plot showed the correlation between the TIME score and the PHLDA2 expression. (H) The correlations between the PHLDA2 and PDCD1. (I) The PHLDA2 expression between parental and sorafenib-resistant group from the GSE121153. *p < 0.05; ***p < 0.001.

CSCs are considered to be the cause of treatment resistance [25,26]. So, we continued to investigate the role of PHLDA2 in the treatment of HCC. We estimated the responsiveness of HCC patients to immunotherapy by ImmuCellAI algorithms. By comparing the expression of PHLDA2 between responder and non-responder, we found that PHLDA2 expression was higher in non-responsive patients (Fig. 6A). We also verified the difference in PHLDA2 expression between responder and non-responder via the TIDE algorithms in the TCGA cohort and the HCCDB6 cohort, respectively (Figs. 6B and 6E). The TIDE score was higher in patients with higher PHLDA2 expression (Fig. 6C), which suggested that these patients are more prone to immune escape. We also found that the MSI score in the PHLDA2-low subgroup was higher (Fig. 6D). In addition, The TIME phenotypes also impact immunotherapy response, according to previous reports [27]. Patients classified as TIME-3 accounted for a higher proportion in the PHLDA2 high subgroup (Fig. 6F). Meanwhile, the TIME score was negatively correlated with PHLDA2 (Fig. 6G). Besides, PHLDA2 was also negatively correlated with PDCD1 (Fig. 6H). These results suggested that patients with low expression of PHLDA2 had a higher effective rate of immunotherapy. In addition, concerning chemotherapy, we compared the expression level of PHLDA2 between the resistant and non-resistant groups in the GSE121153 cohort. We then found that the expression of PHLDA2 was higher in the resistant group, which revealed that PHLDA2 may play a role in sorafenib resistance (Fig. 6I). Finally, we predicted drugs to which patients with high PHLDA2 expression might be sensitive. The result showed that with the increase of PHLDA2 expression, the IC50 of midostaurin, AZ682, pemetrexed, and GNF2 decreased (Suppl. Fig. S3). According to the findings, the treatment effect of patients with lower PHLDA2 expression may be better after chemotherapy and immunotherapy.

### Relevant experimental verification

**Figure 7:**
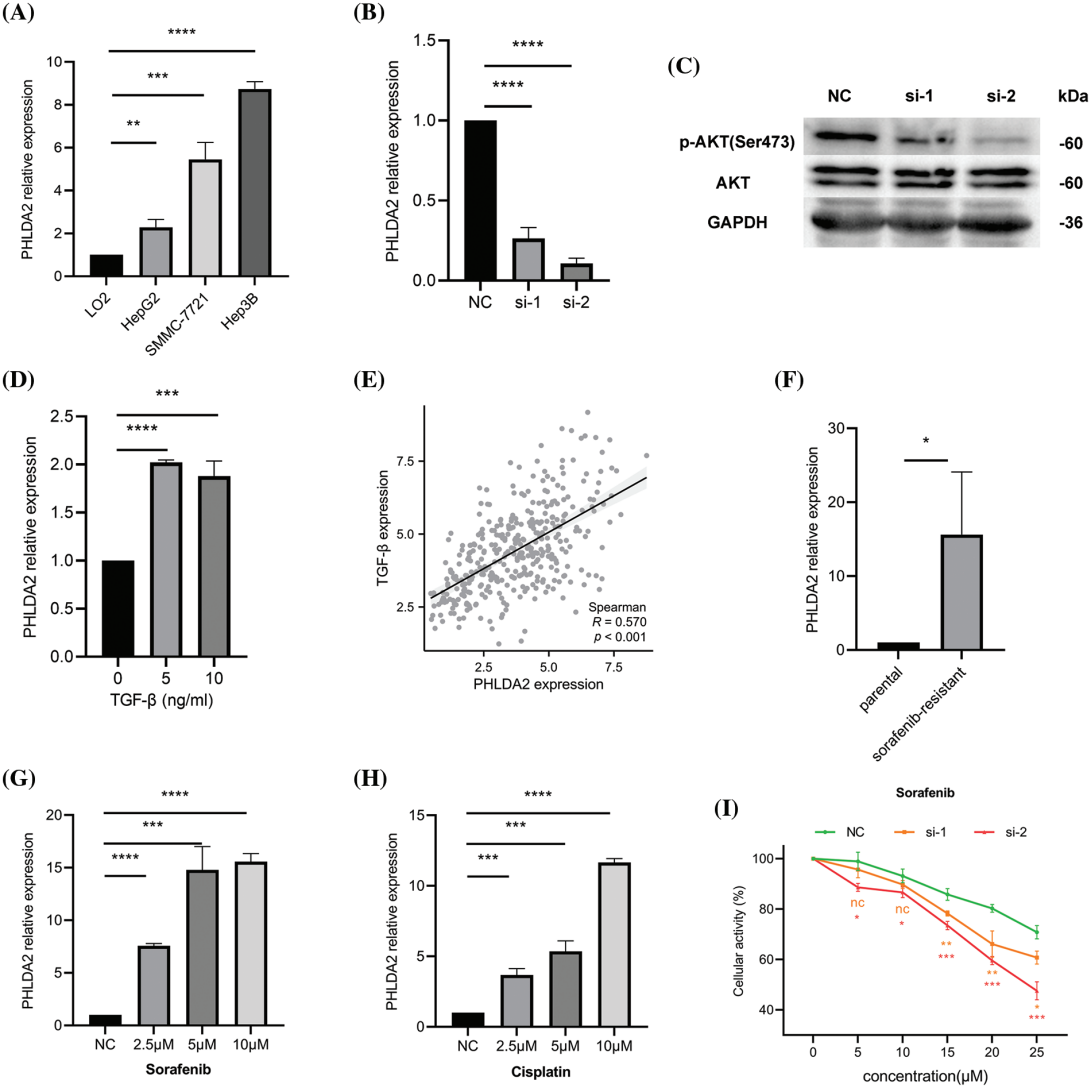
Relevant experimental verification. (A) The expression of PHLDA2 in different cell lines, including LO2, HepG2, SMMC-7721, and Hep3B. (B) qRT-PCR results showed that PHLDA2 had knockdown efficiency after transfection of Hep3B cells with two different siRNAs. (C) WB results revealed p-AKT, AKT, and GAPDH protein expression levels in the NC, si-1, and si-2 groups. (D) The expression of PHLDA2 in Hep3B cells treated with different concentrations of TGF-β. (E) The scatter plot showed the correlation between PHLDA2 and TGF-β in the TCGA database. (F) The expression of PHLDA2 in sorafenib-resistant and parental groups. (G, H) The expression of PHLDA2 in Hep3B cells treated with different sorafenib (G) and cisplatin (H) concentrations. (I) The MTT assay showed the viability of the Hep3B cells after 48 h in gradient doses of sorafenib. *p < 0.05; **p < 0.01; ***p < 0.001; ****p < 0.0001.

Last but not least, we carried out some experiments to verify the results of some bioinformatics analysis. QRT-PCR results demonstrated that PHLDA2 was overexpressed in several HCC cell lines, such as HepG2, Hep3B, and SMMC-7721, compared with the human normal hepatocyte cell lines, LO2 cell (Fig. 7A). Because PHLDA2 had the highest expression in Hep3B cells, we chose Hep3B cells for subsequent experiments. The silencing efficiency of si-RNAs in Hep3B cells is shown in Fig. 7B. After transfection with si-RNAs, the expression of PHLDA2 was significantly decreased. Next, we compared the activity of AKT between si-1, si-2, and NC groups in Hep3B cells. The WB results showed that the Phosphorylated-AKT (p-AKT) protein of si-1 and si-2 groups was decreased (Fig. 7C; Suppl. Fig. S4). In addition, we treated Hep3B cells with TGF-β cytokines. The results showed that the expression of PHLDA2 in cells treated with TGF-β was increased (Fig. 7D). The correlation analysis also showed that PHLDA2 and TGF-β were positively correlated (Fig. 7E). Finally, we investigated whether PHLDA2 would play a role in drug resistance. We compared the expression of PHLDA2 between the sorafenib-resistant and parental groups of Hep3B. The results of qRT-PCR showed that the expression of PHLDA2 was increased in the sorafenib-resistant group (Fig. 7F). In addition, with the increase of sorafenib and cisplatin concentrations, the expression of PHLDA2 also increased (Figs. 7G and 7H). MTT results revealed that the OD490 value ratio was lower in the si-1 and si-2 groups (Fig. 7I). These results indicated that Hep3B is more sensitive to sorafenib after the knockdown of PHLDA2.

## Discussion

Hepatocellular carcinoma (HCC) is a highly prevalent and lethal malignant tumor that poses a significant threat to public health. Recently, there has been a growing interest in exploring the tumor immune microenvironment (TME) and its potential as a target for therapeutic interventions, particularly immunotherapy [28]. However, the lack of reliable biomarkers to assess prognosis and treatment response in HCC patients [29], coupled with the heterogeneous nature of patient responses to existing immunotherapies [30], underscores the urgent need to develop more effective biomarkers. This study has discovered that PHLDA2 induces alterations in the immune microenvironment, exhibits a correlation with cancer stemness and facilitates the development of treatment tolerance.

We presented an integrative analysis of expression, prognosis value, methylation levels, genomic alterations, immune cell infiltration, and potential functions and molecular mechanisms of PHLDA2 in HCC. Our data demonstrated the remarkable upregulation of PHLDA2 expression in HCC. The results of the clinical association analysis indicated the high expression of PHLDA2 indicates larger tumors and later stages in patients. Additionally, high PHLDA2 expression is a factor for predicting adverse prognosis. Consequently, PHLDA2 has the potential to serve as a diagnostic or prognostic biomarker for HCC.

In a broader context, the regulation of gene expression encompasses various mechanisms, such as epigenetic modification, genetic mutations, and transcription factors. Specifically, DNA methylation plays a role in regulating gene expression by impeding the binding of transcription factors to the DNA promoter region [31]. Hypermethylation leads to gene silencing, while hypomethylation results in gene activation [32,33]. Our investigation revealed a decrease in the methylation level of PHLDA2 in HCC, suggesting that the heightened expression of PHLDA2 may be influenced by promoter methylation. Reversal of the hypomethylation may reduce PHLDA2 expression. Next, we proceeded to examine the relationship between PHLDA2 and genomic instability.

Genomic instability, caused by DNA repair gene mutations, is the driving factor of tumor heterogeneity and evolution and could promote cancer development [34,35]. Mutations in various tumor suppressor genes, including TP53 and ATM, have been linked to tumor genomic instability [36,37]. In our study, TP53, acting as a cancer guard, is more susceptible to mutation in HCC patients exhibiting elevated expression of PHLDA2. Furthermore, MATH, LOH, and HRD are the makers of genomic instability. So, we infer that the upregulation of PHLDA2 can increase genomic instability, activate oncogenes, or deactivate tumor suppressor genes, thereby promoting tumor development.

TME, as the “soil” of tumorigenesis, is typically composed of immune cells, stromal cells, ECM, the blood and lymphatic vascular networks, and other secreted molecules [38]. The continuous interaction between various components and tumor cells promotes tumor progression [39]. For example, tumor-associated macrophages (TAMs) can be divided into two subpopulations: anti-tumor M1 macrophages and tumor-promoting M2 macrophages, which can be converted under specific environmental stimuli [40]. M2 macrophages can promote the proliferation, invasiveness, angiogenesis, stemness, and metastasis of HCC in various ways [41,42]. In addition, as immunosuppressive cells, Tregs can inhibit the activity of tumor killer T cells and secrete immunosuppressive molecules, such as TGF-β, to promote the immune escape of tumor cells [43,44]. Therefore, we investigated the involvement of PHLDA2 in the immune microenvironment. Our research revealed that PHLDA2 exhibited the capacity to augment the infiltration of diverse immune cells in HCC, encompassing Tregs, macrophages, dendritic cells, T cells CD4+, T cells CD8+, B cells, and NK cells. Thus, targeting PHLDA2 could potentially impede the recruitment of TAMs, reprogram the polarization of TAMs, and suppress Tregs-induced immune tolerance to enhance anti-tumor immunity, thereby reshaping the tumor immune microenvironment.

Subsequently, GO and KEGG enrichment analysis was conducted on PHLDA2-related genes, revealing significant enrichment in EMT, ECM, cytokine, and chemokine pathways. The outcomes of ssGSEA analysis further confirmed the pivotal involvement of PHLDA2 in the EMT process. PHLDA2 may change the composition of ECM and promote cancer cells to acquire EMT characteristics. The process of EMT is characterized by the acquisition of mesenchymal features by epithelial cells, which is associated with various aspects of tumor development, including initiation, invasion, metabolism, and resistance to therapy [45,46]. Similarly, the dysregulation of ECM, a significant component of the TME, is a remarkable feature of cancer [47]. By targeting PHLDA2, it is possible to reverse the EMT process and improve the dysregulation of the ECM. Furthermore, enrichment analysis revealed that PHLDA2 is primarily involved in the WNT signaling pathway, PI3K-AKT signaling pathway, TNF signaling pathway, and TGF-β signaling pathway. Based on previous research, it has been demonstrated that PHLDA2 plays a role in regulating EMT in colorectal cancer via the PI3K-AKT signaling pathway [15]. Additionally, PHLDA2 has been shown to impact EMT and metastasis in osteosarcoma through the SRC/PI3K/AKT/mTOR and WNT/GSK3-β/β-catenin signaling pathway [14,48,49]. Furthermore, Lu et al. found that the knockdown of PHLDA2 promoted apoptosis and autophagy in glioma through the AKT/mTOR pathway [13]. PHLDA2 may also promotes HCC metastasis and progression through these pathways. In conclusion, it can be inferred that PHLDA2 affects EMT and other phenotypes through the PI3K-AKT and TGF-β signaling pathways.

CSCs possess the functional characteristics of self-renewal and differentiation and an augmented capacity for therapeutic resistance, immune evasion, invasion, and metastasis [50]. The stemness of tumor cells can be influenced by various factors, such as EMT, ECM, and various immune cells [51–53]. Our research indicated a potential association between PHLDA2 and the maintenance of stemness in HCC. The clustering analysis revealed distinct clusters exhibiting diverse levels of stemness. Notably, PHLDA2 expression consistently exhibited the highest levels within clusters characterized by the highest stemness. Furthermore, PHLDA2 positively correlated with various molecules, such as CD24, CD47, EPCAM, and ICAM1. These molecules play important roles in the maintenance of cancer stemness [54–56]. The targeting of PHLDA2 may lead to a reduction in the expression levels of these molecules, thereby attenuating stemness. In summary, PHLDA2 exhibits a significant association with tumor stemness.

The cancer-stem-cell model provides an explanation for various clinical phenomena observed in cancer, such as tumor recurrence after successful chemotherapy and radiotherapy, tumor dormancy, and treatment resistance [52]. Systemic therapy, including chemotherapy and immunotherapy, is recommended for advanced HCC patients [57]. The first-line treatment options for these patients include atezolizumab + bevacizumab, sorafenib, and lenvatinib [58]. However, it should be noted that while sorafenib is the initial drug employed to enhance the prognosis of HCC patients, not all individuals will derive benefits from it due to the drug resistance. Numerous prior studies have investigated the underlying mechanisms of sorafenib resistance, such as TME, EMT, and stemness enhancement in HCC [26,59]. Occasionally, sorafenib-resistant HCC cells exhibit a notable EMT phenotype and possess stemness characteristics [26,60]. Several studies have indicated that the effectiveness of sorafenib treatment can be enhanced by inhibiting EMT and weakening stemness in HCC [61,62]. A study conducted by Chang et al. has demonstrated that modulating the TME by targeting tumor infiltrating Ly6G^+^ myeloid cells is a potential strategy to enhance sorafenib efficacy [63]. Zhou et al. revealed tumor-associated neutrophils could recruit macrophages and Tregs into TME to promote sorafenib resistance [64]. Consequently, it is crucial to identify novel targets to overcome sorafenib resistance. In our research, PHLDA2 was associated with EMT and stemness and was involved in the modulation of TME. Therefore, we suspected that PHLDA2 may contribute to sorafenib resistance in HCC. As expcetion, our findings confirmed that PHLDA2 was elevated in both non-responders to immunotherapy and sorafenib-resistant patients. This can provide new ideas for the treatment of liver cancer.

To verify the aforementioned analytical outcomes, we conducted relevant experiments. We first confirmed the high expression of PHLDA2 in HCC cells through *in vitro* experiments. Based on the findings of the enrichment analysis, our experimental results have substantiated that the downregulation of PHLDA2 leads to a reduction in AKT phosphorylation levels. PHLDA2 may exert regulatory control over the AKT signaling pathway, thereby facilitating the advancement of hepatocellular carcinoma (HCC). Moreover, our observations demonstrate an upregulation of PHLDA2 expression upon treatment with TGF-β, thereby indicating its involvement in the TGF-β signaling pathway. Additionally, we subjected Hep3B cells to varying concentrations of sorafenib and cisplatin for further investigation. Our findings indicate that PHLDA2 showed its responsiveness to sorafenib during the acute phase of treatment. Additionally, the expression of PHLDA2 was elevated in sorafenib-resistant Hep3B cells. Furthermore, we observed that knocking of PHLDA2 weakened the resistance of HCC cells to sorafenib by the MTT assay, indicating the involvement of PHLDA2 in sorafenib resistance.

In summary, our study revealed the role of PHLDA2 in HCC. However, this study also has certain limitations. Firstly, the data used for analysis needs to be more comprehensive. More clinical data should be included, and the prognostic value of PHLDA2 should be verified in more cohorts and populations. Secondly, more experiments are needed to explore the specific pathways and mechanisms in which PHLDA2 is involved. As for the role of PHLDA2 on immune regulation, more basic research is required in the future.

In conclusion, PHLDA2 is highly expressed in HCC, and high expression levels of PHLDA2 increase immune cell infiltration, enhance stemness, and induce drug resistance. Consequently, targeting PHLDA2 emerges as a potential therapeutic approach for managing HCC.

## Supplementary Materials

FIGURE S1. The correlation between PHLDA2 and B cells, DC cells, NK cells, T cells CD8+, T cells CD4+, endothelial cells.

FIGURE S2. The correlation between PHLDA2 and immune cells from the TISCH database.

FIGURE S3. The correlation between PHLDA2 and IC50 of drugs.

FIGURE S4. Western blots of p-AKT, AKT, and GAPDH

## Data Availability

The datasets used and analyzed during the current study are available from the corresponding author upon reasonable request. Any additional information required to reanalyze the data reported in this paper is available from the lead contact upon request.

## Supplementary Materials

The supplementary material is available online at https://doi.org/10.32604/or.2024.047078.
