# Supplementary figures and images for: PHLDA2 reshapes the immune microenvironment and induces drug resistance in hepatocellular carcinoma
Source: Oncol Res. 2024 May 23;32(6):1063–78. doi: 10.32604/or.2024.047078 (PMC11136693; doi:10.32604/or.2024.047078)

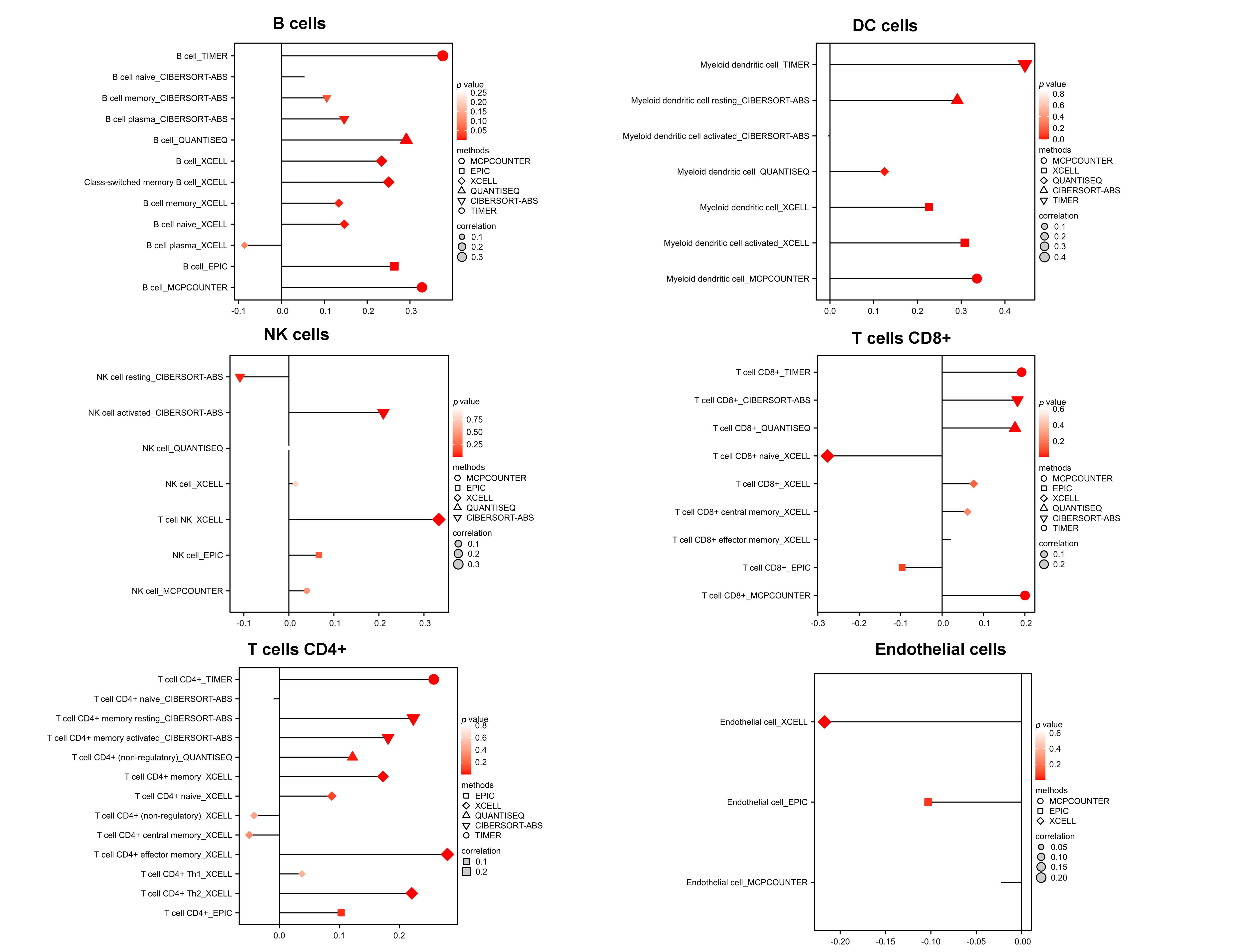

Supplement: FIGURE S1. [file OncolRes-32-47078-s001.tif]

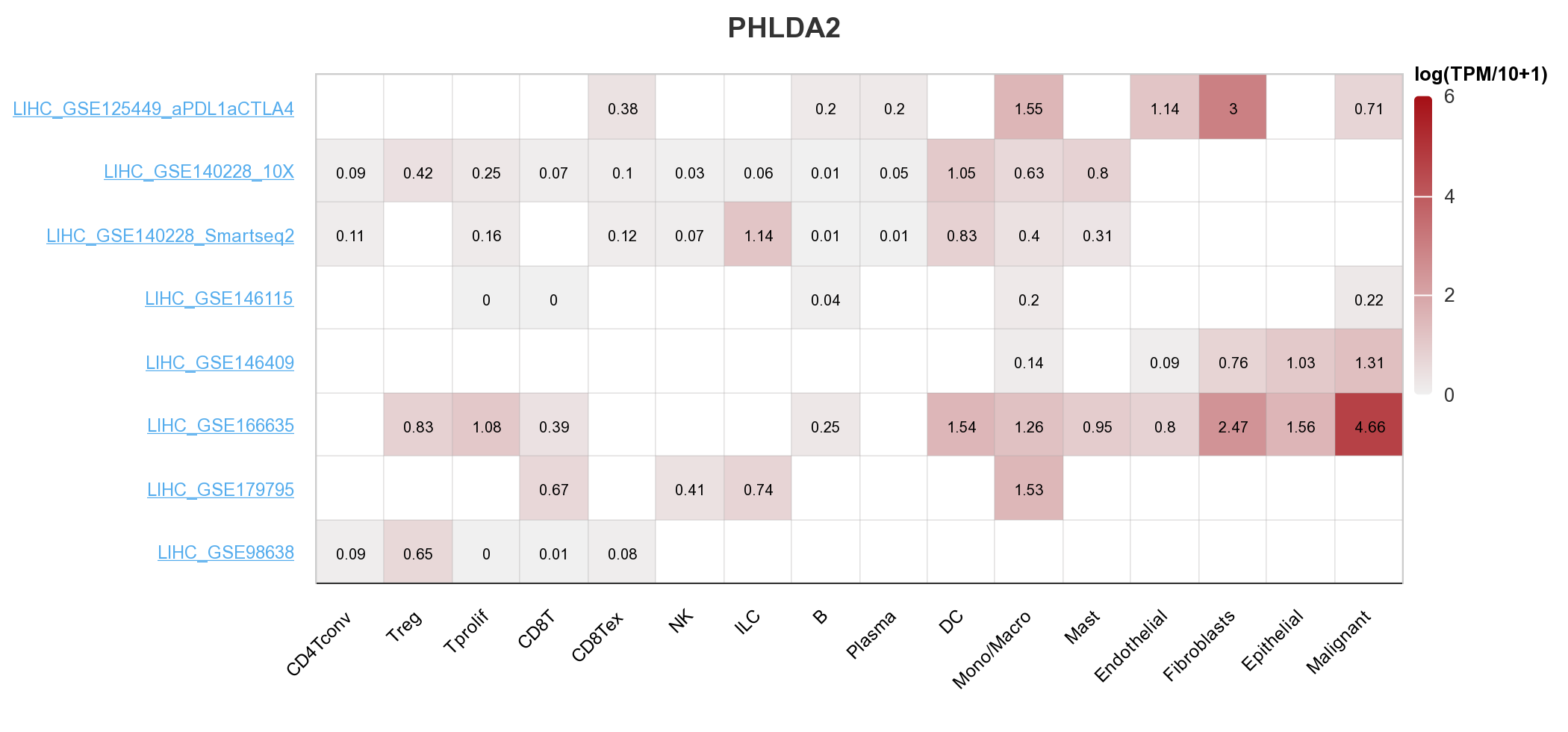

Supplement: FIGURE S2. [file OncolRes-32-47078-s002.tif]

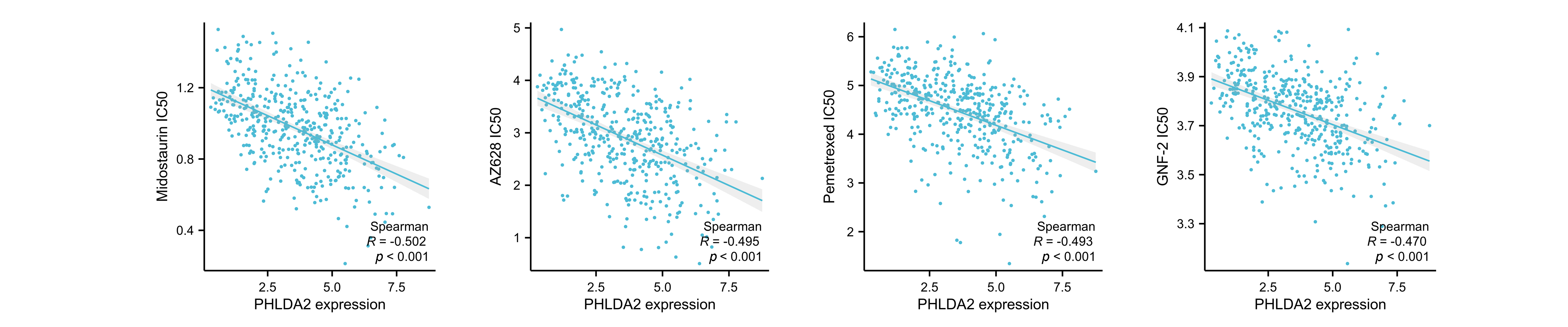

Supplement: FIGURE S3. [file OncolRes-32-47078-s003.tif]

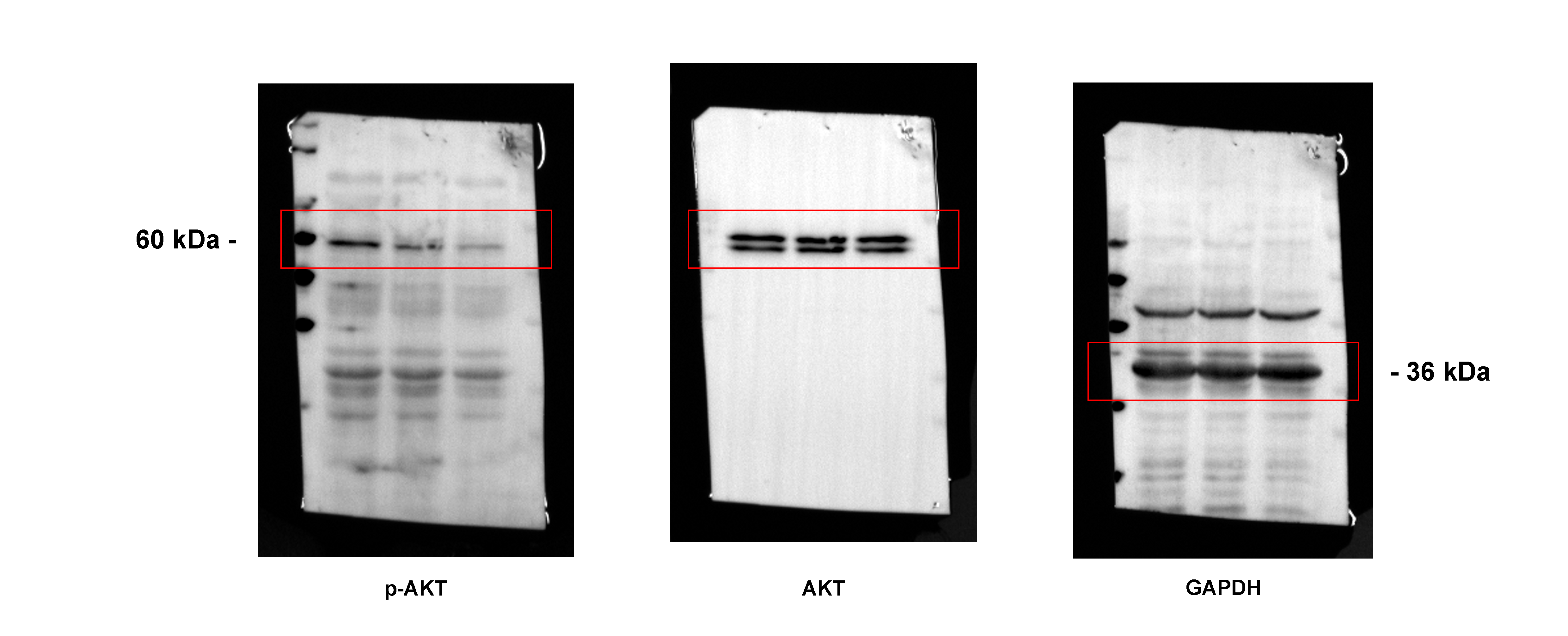

Supplement: FIGURE S4. [file OncolRes-32-47078-s004.tif]
